# Release of Sequestered Malaria Parasites upon Injection of a Glycosaminoglycan

**DOI:** 10.1371/journal.ppat.0020100

**Published:** 2006-09-29

**Authors:** Anna M Vogt, Fredrik Pettersson, Kirsten Moll, Cathrine Jonsson, Johan Normark, Ulf Ribacke, Thomas G Egwang, Hans-Peter Ekre, Dorothe Spillmann, Qijun Chen, Mats Wahlgren

**Affiliations:** 1 Department of Microbiology, Tumor, and Cell Biology, Karolinska Institutet, Stockholm, Sweden and Swedish Institute for Infectious Disease Control, Solna, Sweden; 2 Department of Nuclear Medicine, Karolinska University Hospital, Solna, Sweden; 3 Med Biotech Laboratories, Kampala, Uganda; 4 Dilafor AB, Stockholm, Sweden; 5 Department of Medical Biochemistry and Microbiology, Uppsala University, Uppsala, Sweden; Stanford University, United States of America

## Abstract

Severe human malaria is attributable to an excessive sequestration of Plasmodium falciparum–infected and uninfected erythrocytes in vital organs. Strains of P. falciparum that form rosettes and employ heparan sulfate as a host receptor are associated with development of severe forms of malaria. Heparin, which is similar to heparan sulfate in that it is composed of the same building blocks, was previously used in the treatment of severe malaria, but it was discontinued due to the occurrence of serious side effects such as intracranial bleedings. Here we report to have depolymerized heparin by periodate treatment to generate novel glycans (dGAG) that lack anticoagulant-activity. The dGAGs disrupt rosettes, inhibit merozoite invasion of erythrocytes and endothelial binding of P. falciparum–infected erythrocytes in vitro, and reduce sequestration in in vivo models of severe malaria. An intravenous injection of dGAGs blocks up to 80% of infected erythrocytes from binding in the micro-vasculature of the rat and releases already sequestered parasites into circulation. P. falciparum–infected human erythrocytes that sequester in the non-human primate Macaca fascicularis were similarly found to be released in to the circulation upon a single injection of 500 μg of dGAG. We suggest dGAGs to be promising candidates for adjunct therapy in severe malaria.

## Synopsis

Severe Plasmodium falciparum malaria is common and in part the result of an excessive binding of infected erythrocytes in the microvasculature. The parasite employs heparan sulfate during the adherence to the vascular endothelium and to erythrocytes. Heparin, which is related to heparan sulfate in that it is composed of the same building blocks, was here periodate-treated to generate depolymerized glycosaminoglycans (dGAGs) that possess no anticoagulant activity. The dGAGs disrupt erythrocyte and endothelial binding of P. falciparum–infected erythrocytes in vitro. An intravenous injection of dGAGs blocks infected erythrocytes from binding in the micro-vasculature of the rat and releases already sequestrated parasites into circulation both in the rat and in a non-human primate. If this approach is successfully translated to the clinical setting, it may offer help to patients whereby the injection of a dGAG releases already sequestered parasite-infected erythrocytes and re-establishes the micro-vascular blood flow. The authors suggest dGAGs to be promising candidates of adjunct therapy that may have an important impact on malaria mortality.

## Introduction

During Plasmodium falciparum malaria, signs of severe anemia, respiratory distress, and cerebral malaria, or combinations thereof, are common. These disease states are in part a result of the excessive binding of P. falciparum–infected erythrocytes (IE) to the vascular endothelium (cytoadherence), and to infected and uninfected erythrocytes (rosetting, giant-rosetting, and auto-agglutination). The sequestration may lead to occlusion of the microvasculature, which thereby contributes directly to the acute pathology of severe human malaria [1–5].

The mechanisms behind sequestration of IE are to a large degree dependent on the interaction between P. falciparum erythrocyte membrane protein 1 (PfEMP1), a parasite-derived molecule present at the surface of the IE [6,7], and host receptors on endothelial and red blood cells [8–15]. PfEMP1 is composed of multiple cystein-rich extracellular domains, which show affinity for an array of host receptors including the glycoasaminoglycan (GAG) heparan sulfate (HS) [12,16–20]. The parasite employs HS during the adherence both to the endothelium and to erythrocytes [12,16–20]. Binding to the HS receptor is mediated by the N-terminal Duffy-binding-like domain 1α (DBL1α) of PfEMP1 in which high-affinity binding requires 12-mers of HS as well as the presence of *N-*, 2-, and 6-*O*-sulfate groups [18,21]. HS and heparin inhibit and reverse cytoadherence and rosetting of laboratory strains and wild isolates in vitro [3,12,16–19]. Furthermore, the capacity of IE to adhere to HS is more frequent in isolates of children with severe disease than in those with mild malaria, suggesting HS to be one of the sequestration receptors participating in the causation of severe malaria [4].

Heparin, which is related to HS in that it is composed of the same building blocks (glucosamine and glucuronic or iduronic acid) and is negatively charged through sulfate groups, was previously used in the adjunct treatment of severe malaria with overall successful outcomes [22–26], but its use was discontinued due to the occurrence of serious side effects such as intracranial bleedings [27]. A rare pentameric sequence present in standard heparin has high affinity for antithrombin III (AT) and is responsible for its anticoagulant activity [28]. As heparin is known to be susceptible to selective oxidation at this specific pentameric sequence [29], we periodate-treated heparin, generating depolymerized glycosaminoglycans (dGAGs) that possess no anticoagulant activity, but otherwise still is similar to HS/heparin. dGAGs are here shown to have the same effect as heparin and HS in IE adhesion experiments in vitro, and it is demonstrated to have antisequestration effects in vivo, employing two newly developed animal models in rats and monkeys.

## Results

### Generation and Evaluation of dGAG

Two batches of dGAGs (KI01 and DFX-101) were generated by periodate treatment of heparin as described under Materials and Methods; one batch (DFX-101) was created under GMP conditions to allow administration to primates. The preparations were evaluated as to anticoagulant activity and the capacity to bind to live IE and to recombinant PfEMP1. The anticoagulant activities of the two dGAG preparations (KI01 and DFX-101), un-fractionated (standard) heparin, AT affinity–selected heparin (high and low AT–affinity fractions), and buffer, were evaluated using an APTT (activated partial thromboplastin time) test. This test is sensitive to factors XII, XI, IX, VIII, X, V, and II, but insensitive to factors VII and XIII, and measures the overall activity of the intrinsic coagulation system. Plasma samples clotted within minutes after the addition of dGAG-KI01, dGAG-DFX-101, low AT–affinity heparin, or buffer, whereas the samples did not clot even after 2 h if treated with standard heparin or high AT–affinity heparin (Table 1).

**Table 1:**
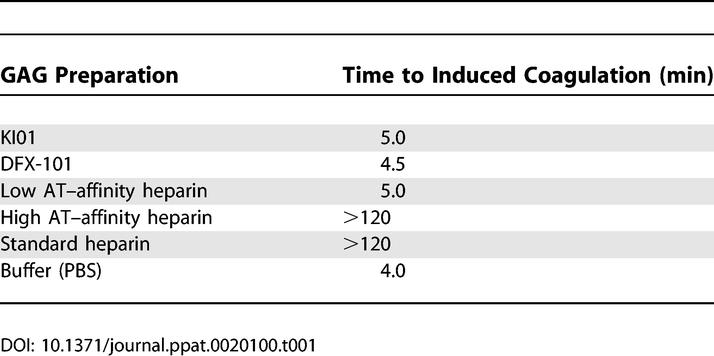
Anticoagulant Activity of Different GAG-Preparations Measured with APTT Test (Cephotest)

**Figure 1:**
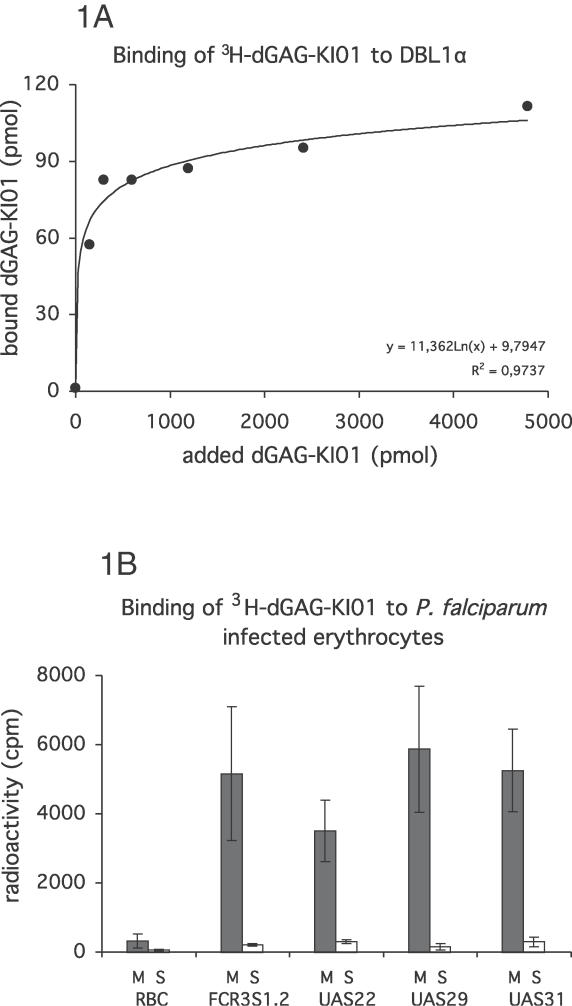
Binding of dGAG-KI01 to P. falciparum Antigens (A) dGAG-KI01 binding to DBL1α was analyzed using an in-solution assay. A fixed concentration (4 μg/ml) was allowed to incubate with increasing concentrations of ^3^H-dGAG-KI01. Protein with bound dGAG was recovered by membrane filtration. (B) Aliquots of rosetting parasite cultures (UAS22, UAS29, UAS31, and FCR3S1.2) were incubated with ^3^H-dGAG-KI01. Cells were lysed in hypotonic buffer, and membranes (M) were separated from the supernatants (S). ^3^H-dGAG-KI01 bound to DBL1α and cell compartments were analyzed by scintillation counting.

dGAG-KI01 was radiolabeled and further tested for binding to recombinant DBL1α, the known HS- and heparin-binding domain of PfEMP1 [12,19], and for binding to live IE. Recombinant DBL1α-GST fusion protein was expressed and purified, and its binding capacity to ^3^H-labeled dGAG-KI01 was assessed in an in-solution assay. A κ_D_ of approximately 0.3 μM was estimated for the binding of dGAG-KI01 to DBL1α-GST (Figure 1A), whereas no binding was seen to GST alone (unpublished data). The results were confirmed by letting the dGAG interact with intact, live P. falciparum–infected erythrocytes at the trophozoite stage in which the protein antigens are presented at the cell surface with the native folding. ^3^H-labeled dGAG-KI01 was incubated with IE of FCR3S1.2, of IE from three recently established clinical isolates of Ugandan children with severe malaria (UAS22, UAS29, and UAS31), or with uninfected erythrocytes. Membranes and supernatants were separated and analyzed for ^3^H-dGAG-KI01 by scintillation counting (see also [19]). dGAG-KI01 was demonstrated to interact with the membranes of IE of the FCR3S1.2 and of the Ugandan isolates UAS22, UAS29, and UAS3, but not with the membranes of uninfected erythrocytes (Figure 1B). ^3^H-dGAG-KI01 did not pass the erythrocyte membrane, whether infected or not, as no ^3^H was found in any of the supernatants (Figure 1B).

### Effects of dGAGs on Rosetting and Cytoadherence In Vitro

**Figure 2:**
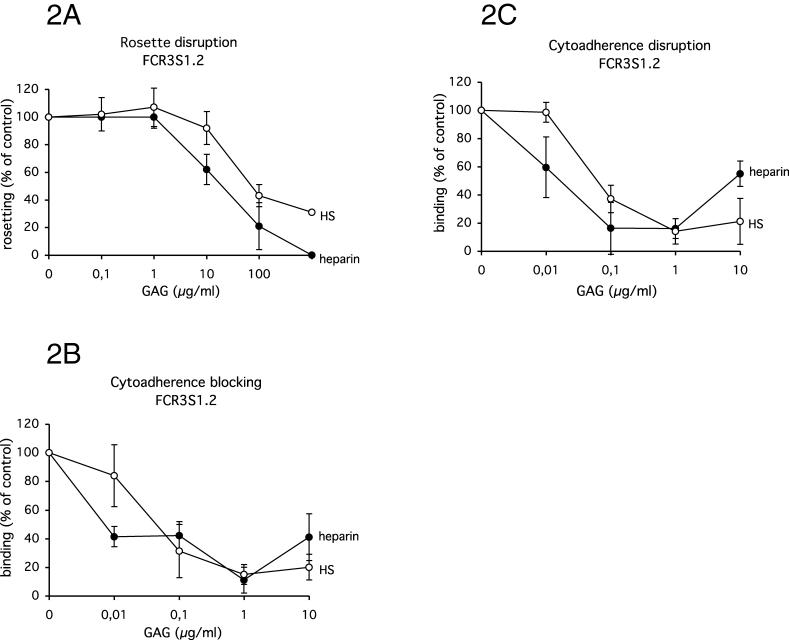
Effect of Heparin and HS on P. falciparum Rosettes and Cytoadherence of IE In Vitro (A) Aliquots of rosetting parasite cultures (FCR3S1.2) were treated with heparin or HS at different concentrations. The rosetting-rates were counted after 30 min incubation and compared with mock treated samples. The cytoadherence assays (B and C) with IE (FCR3S1.2) and heparin or HS were performed under orbital shaking (50 rpm) at 37 °C. Increasing concentrations of heparin and HS were added together with IE (B) or after letting the IE adhere (C). Unbound material was removed before the slides were fixed in glutaraldehyde, stained with Giemsa and analyzed by light microscopy at a x1,000 magnification.

**Figure 3:**
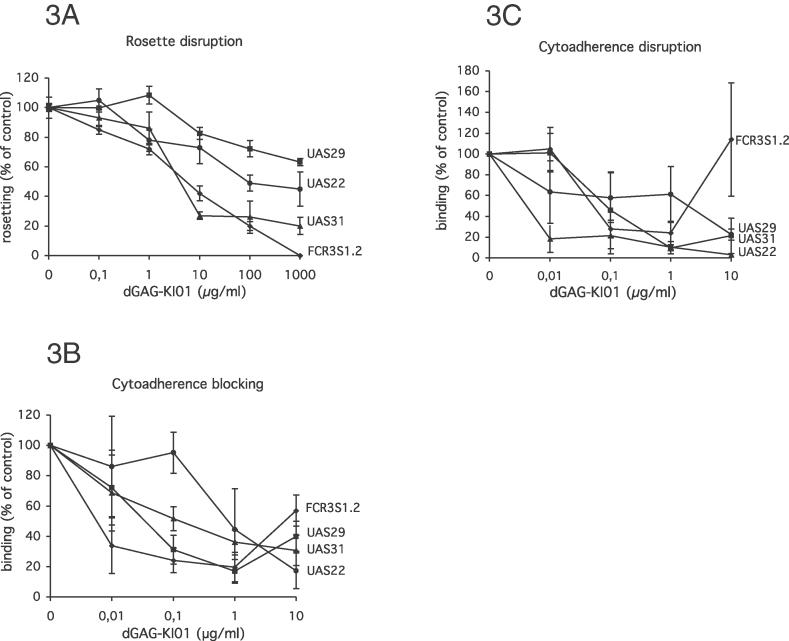
Effect of dGAG-KI01 on P. falciparum Rosettes and Cytoadherence of IE In Vitro (A) Aliquots of rosetting parasite cultures (UAS22, UAS29, UAS31, and FCR3S1.2) were treated with dGAG-KI01 at different concentrations. The rosetting rates were counted after 30-min incubation and compared with mock-treated samples. (B) and (C) For the cytoadherence assays, different P. falciparum IE (UAS22, UAS29, UAS31, and FCR3S1.2) were allowed to attach to rat lung sections under orbital shaking (50 rpm) at 37 °C. Different concentrations of dGAG-KI01 were added together with IE (B) or after letting the IE adhere (C). Unbound material was removed by washes before the slides were fixed in glutaraldehyde, stained with Giemsa, and analyzed by light microscopy at a 1,000× magnification.

Rosetting and cytoadherence of the highly rosetting and multi-adhesive clone FCR3S1.2 are sensitive to heparin and HS [12,16–20] as shown in Figure 2A–2C. FCR3S1.2 as well as the UAS22, UAS29 and UAS31 clinical isolates were furthermore tested for their sensitivity to dGAGs in rosetting and cytoadherence assays. Both dGAG-KI01 and dGAG-DFX-101 disrupted rosettes of all parasite cultures in a dose-dependent manner and total or close to total disruption was reached at 1 mg/ml with rosettes of FCR3S1.2 and UAS31 (Figure 3A and Figure S1). The rosettes of the clinical isolates UAS22 and UAS29 were less sensitive to dGAG-KI01 and dGAG-DFX-101 (Figure 3A and Figure S1).

The effect of dGAG-KI01 on the binding of IE to cryosections of rat lung was evaluated by dynamic incubation in order to mimic in vivo blood flow conditions. The direct effect on primary binding to rat lung sections was examined by adding enriched IE together with dGAG simultaneously to the lung sections (cytoadherence blocking). Up to 80% of the binding of IE was blocked by dGAG-KI01 as compared with untreated samples (Figure 3B). The blocking of binding of FCR3S1.2 and UAS29 IE was less effective at concentrations of 10 μg/ml than at 1 μg/ml. In order to test the efficiency of the dGAGs to dislodge already bound IE from rat lung sections, IE were allowed to adhere to the lung sections, and unbound IE were washed off before incubation with the dGAGs at different final concentrations (cytoadherence disruption). Cytoadherence disruption with dGAG-KI01 resulted in up to 100% reduction of binding (Figure 3C). The effect of dGAG-DFX-101 was also evaluated and found to be comparable to the effect of dGAG-KI01, both when blocking or disrupting the cellular interactions (Figure S1).

### Effects of dGAGs on Merozoite Invasion of Erythrocytes In Vitro

**Figure 4:**
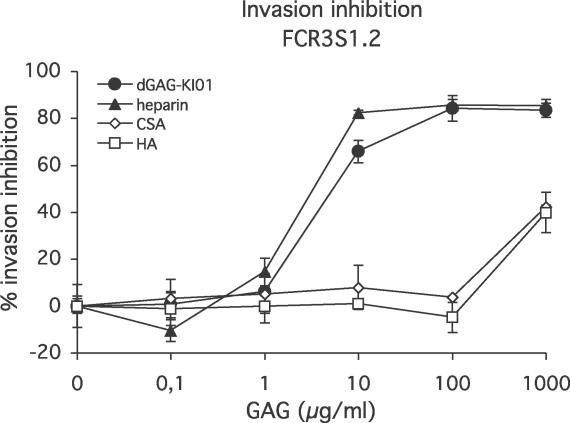
Effects of Different GAGs on Reinvasion of P. falciparum Merozoites (FCR3S1.2) into Fresh Erythrocytes In Vitro Parasite culture at throphozoite stage (≈25 h of development) at a 0.4% parasitemia and a 2% hematocrit were incubated with increasing concentrations of dGAG-KI01, heparin, CSA, or HA for 24–30 h at 37 °C. Parasitemias were estimated using FACS counting with a minimum of 50,000 cells per sample.

Heparin has previously been demonstrated to inhibit continuous cultivation of P. falciparum in vitro by blocking merozoite invasion of erythrocytes [30–32]. dGAG-KI01, dGAG-DFX101, standard heparin, chondroitin sulfate A (CSA), and hyaluronic acid (HA) were therefore tested for their blocking effects on merozoite invasion of erythrocytes using an in vitro assay. dGAG–KI01 and dGAG-DFX-101 blocked merozoite invasion in a dose-dependent manner and reached up to 83% and 86% inhibition, respectively (Figure 4 and Figure S2). The inhibitory effects of the dGAGs were found to be equal to those of standard heparin (Figure 4). CSA, which is similar to heparin and dGAG as it is a negatively charged, sulfated molecule, and HA, a negatively charged but non-sulfated molecule, showed only small effects on merozoite invasion at the highest concentration tested (41% and 40% inhibition, respectively), whereas there was no effect at lower concentrations (Figure 4).

### Effect of dGAGs on Sequestration of IE in Rats In Vivo

**Figure 5:**
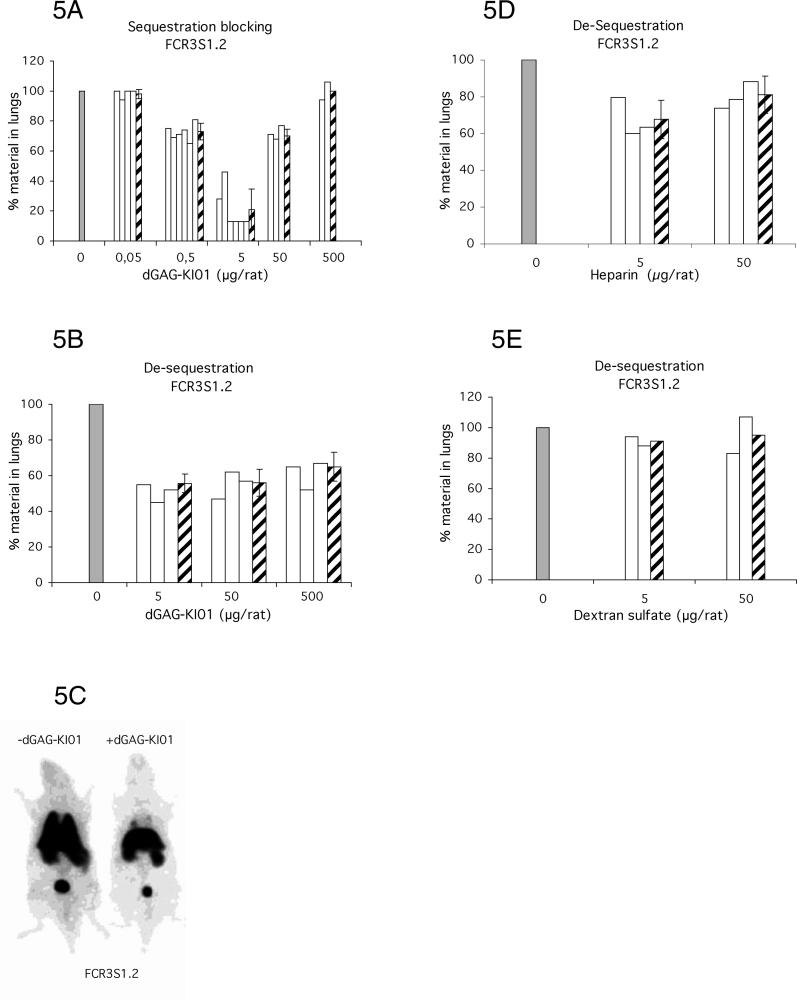
Injection of dGAG-KI01 into Rats Prevents Sequestration of P. falciparum (FCR3S1.2) IE in the Lungs (A) and (B) Rats received either ^99m^Tc-labeled IE simultaneously with different concentrations of the dGAG-KI01 (sequestration blocking) (A), or were first injected with ^99m^Tc-labeled IE and, after three min, injected with dGAG-KI01 (de-sequestration) (B). (C) Whole-body summary images of rats captured 21–30 min after the injection of ^99m^Tc-labeled IE alone (−dGAG-KI01) or when co-injected with 5 μg of dGAG-KI01 (+dGAG-KI01). (D) and (E) Rats were injected with ^99m^Tc-labeled IE and subsequently with heparin (D) or the sulfated polysaccharide dextran sulfate (E). Rats were in all cases left in the gamma camera for 30 min after which the lungs were excised, measured for radioactivity, and compared to the level of radioactivity in the whole animal. Results are presented as relative amount in lungs compared with control animals receiving no dGAG-KI01, heparin, or dextran sulfate (control, adjusted to 100%; grey bars). White bars show the results of individual rats, and striped bars, the mean thereof.

To evaluate whether the dGAGs were able to block and reverse binding of IE in a more complex system, we explored a recently developed animal model for studies of IE sequestration, including both rosetting and cytoadherence [33]. In this in vivo system it has been demonstrated that *P. falciparum–*infected human erythrocytes of different strains and clones robustly adhere in the rat lungs in a PfEMP1-dependent manner [33]. Two different experimental set-ups were used in which IE (FCR3S1.2) were either mixed with dGAGs prior to injection into the rats in order to test the effect on blocking primary binding (sequestration blocking) or the dGAGs were injected after the establishment of IE-sequestration (sequestration disruption). The sequestration of IE in the lungs was 5.2% for FCR3S1.2 of the total amount of material injected. The sequestration-blocking effect of the dGAGs showed a complex but dose-dependent effect. The best effect was seen with the intermediate quantity of dGAG-KI01 showing an approximately 80% average reduction of sequestration at 5-μg dGAG-KI01 injected/animal, whereas an approximately 30% reduction of sequestration at 0.5 and 50 μg/animal and no, or almost no, effect at 0.05 and 500 μg/animal were seen (Figure 5A and 5B). Co-injection of uninfected human erythrocytes with dGAG-KI01 was compared with injection of uninfected erythrocytes without dGAG-KI01. No difference was seen, and the amount was stable below 1% (unpublished data).

Rats were also treated with GAGs *after* the IE (FCR3S1.2) had sequestered in order to study the capacity of the GAGs to release IE into circulation. At 5-μg dGAG injected/animal, sequestration was reduced by approximately 50%, and a similar effect was seen at 50 μg (Figure 5C and Figure S3). When standard heparin was injected, the sequestration decreased somewhat less than after injection of dGAGs since at 5-μg heparin/rat, sequestration was reduced by approximately 30% (Figure 5D). The lower effect of heparin than of dGAG could be due to the capacity of heparin to not only bind to DBL1α, but also to AT, mediating the anticoagulant activity. Further, during surgery for removal of the lungs, it was observed that the blood did not coagulate as in dGAG-treated animals. An injection of dextran sulfate, an unrelated negatively charged, sulfated polysaccharide did not result in a significant reduction of the sequestration (Figure 5E). Mock treatment of animals carrying bound IE with RPMI-1640 also did not affect the sequestration (unpublished data).

### Effect of dGAG on Sequestration of IE of Clinical Isolates in Rats

**Figure 6:**
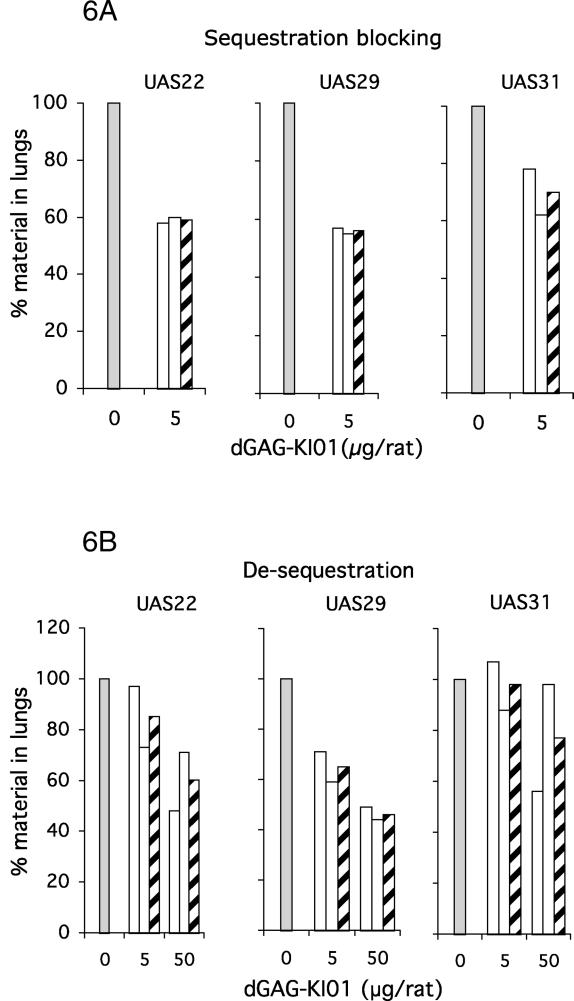
Injection of dGAG-KI01 Decreases P. falciparum Sequestration of IE of Clinical Isolates in the Rat (A) Rats were co-injected with ^99m^Tc-labeled IE (clinical isolates: UAS22, UAS29, and UAS31) and 5 μg of the dGAG-KI01, or (B) rats were first administrated with ^99m^Tc-labeled IE (clinical isolates: UAS22, UAS29, and UAS31) and after 3 min, injected with different concentrations of the dGAG-KI01. Rats were in all cases left in the gamma camera for 30 min, after which the lungs were excised, measured for radioactivity, and compared to the level of radioactivity in the whole animal. Results are presented as relative amount in lungs compared with control animal receiving no dGAG-KI01 (control, adjusted to 100%; grey bars). White bars show the results of individual rats, and the striped bars, the mean thereof.

The effect of dGAG-KI01 and dGAG-DFX-101 on sequestration of IE of three clinical isolates of Ugandan children with severe malaria (UAS22, UAS29, and UAS31; for status of parasites, see Table 2) was also studied in the rat model. Since the material was limited, the experiments could be performed only in duplicates and with fewer concentrations of the dGAGs (5 μg injected/animal for blocking of sequestration, and 5 and 50 μg injected/animal for de-sequestration). Two or three control animals that did not receive any dGAG were run in parallel during each experiment. Lung sequestration was 1.8% for UAS22 (one experiment, two animals), 4.0% for UAS29 (three experiments, six animals), and 10.5% for UAS31 (one experiment, three animals) of the total amount of material injected. Blocking of sequestration with 5 μg/animal dGAG-KI01 or dGAG-DFX-101 resulted in a reduced binding of the IE of all three isolates, but the levels of inhibition varied between the isolates (Figure 6A and Figure S3). Rats treated with dGAG-KI01 or dGAG-DFX-101 *after* the IE had sequestered showed substantial reduction of sequestration when injected with 50 μg/animal, but with 5 μg/animal of dGAGs, moderate effects on the sequestration were seen (Figure 6B and Figure S3).

**Table 2:**
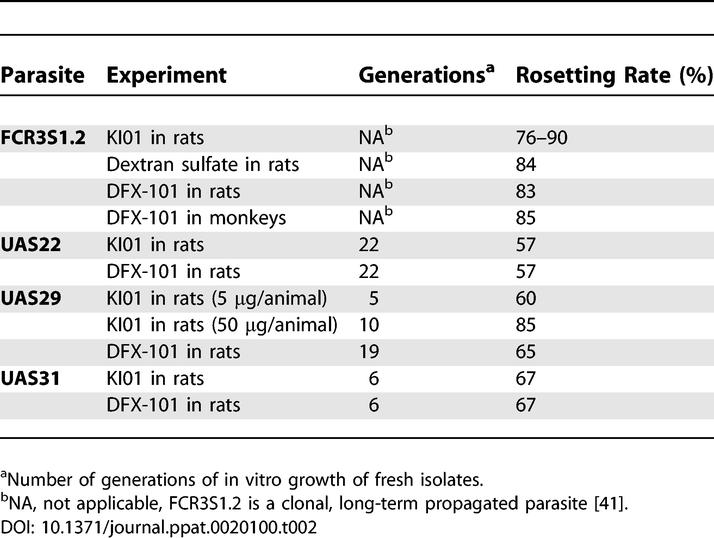
Rosetting Rates and Number of Generation of In Vitro Growth of Parasites Used in the Animal Experiments

### Sequestration of P. falciparum–Infected Erythrocytes in Macaca fascicularis

**Figure 7:**
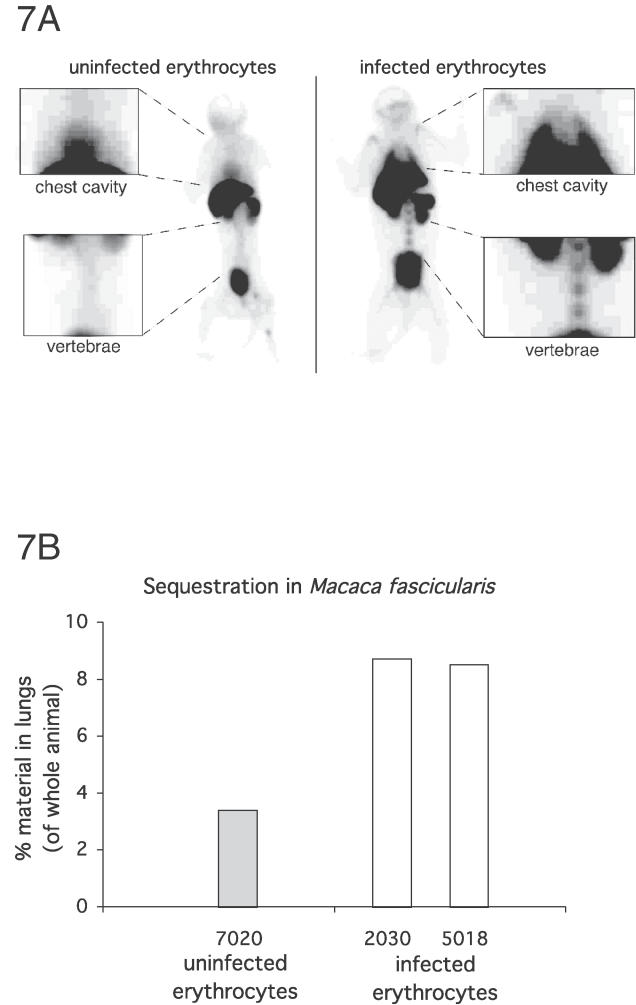
Sequestration of P. falciparum IE of Parasite FCR3S1.2 in Macaca fascicularis Monkeys were injected with ^99m^Tc-labeled IE of FCR3S1.2 or with ^99m^Tc-labeled uninfected human erythrocytes and left for 60 min followed by analysis in an X-ray–equipped triple-headed gamma camera (ID 7020, uninfected erythrocytes; ID 2030 and 5018, IE). (A) Shows the whole-body summary images captured 60–75 min after the injection of the ^99m^Tc-labeled cells. Amplified areas show the chest cavities and the vertebrae. The accumulation of ^99m^Tc-labeled IE in the lungs and in the bone marrow suggests their specific sequestration. The activity in the heart of the animal that received uninfected erythrocytes indicates that the erythrocytes are in circulation. (B) Shows the relative amount of material in the lungs as compared with the activity of the whole animal and measured for 15 min (60 to 75 min after injection). White bars show results of individual monkeys injected with IE, whereas the grey bars show the results of the control animal injected with uninfected human erythrocytes.

A new animal model for the study of the sequestration of human P. falciparum–infected erythrocytes in macaques *(M. fascicularis)* was developed based on the rat model used herein [33]. Briefly, sedated macaques were administrated with approximately 10^8^ highly purified ^99m^Technetium (^99m^Tc)-labeled human uninfected erythrocytes or *P. falciparum–*infected erythrocytes of the FCR3S1.2 clone by injection into the *Vena saphena magna.* The animals were left for 30 min (time point 30 min) to allow IE to sequester and thereafter either kept untreated or treated with dGAG-DFX-101 and left for an additional 30 min (time point 60 min). Macaques were in a first set of experiments examined for the identification of sites where sequestration occurred. At time point 30 min, one of the animals (ID 5018) injected with IE (FCR3S1.2) showed 9.4% of the injected material in the lungs. At time point 60 min, the proportion of material in the lungs was 8.5% (ID 5018) and 8.7% (ID 2030, not measured at 30 min), indicating sequestration in the lungs to be stable (Figure 7). Binding of ^99m^Tc-labeled IE was also seen in the bone marrow of the vertebrae and the humerus (Figure 7A). A total of 3.4% of the material was detected in the lungs of an animal (ID 7020) injected with ^99m^Tc-labeled uninfected erythrocytes, and a large fraction of the radioactivity was also found in the heart and therefore in circulation (Figure 7). Taken together, this indicates that specific sequestration of human IE occurs primarily in the lungs of M. fascicularis as previously observed in the rat [33].

### Effect of dGAG on IE Sequestration in M. fascicularis

**Figure 8:**
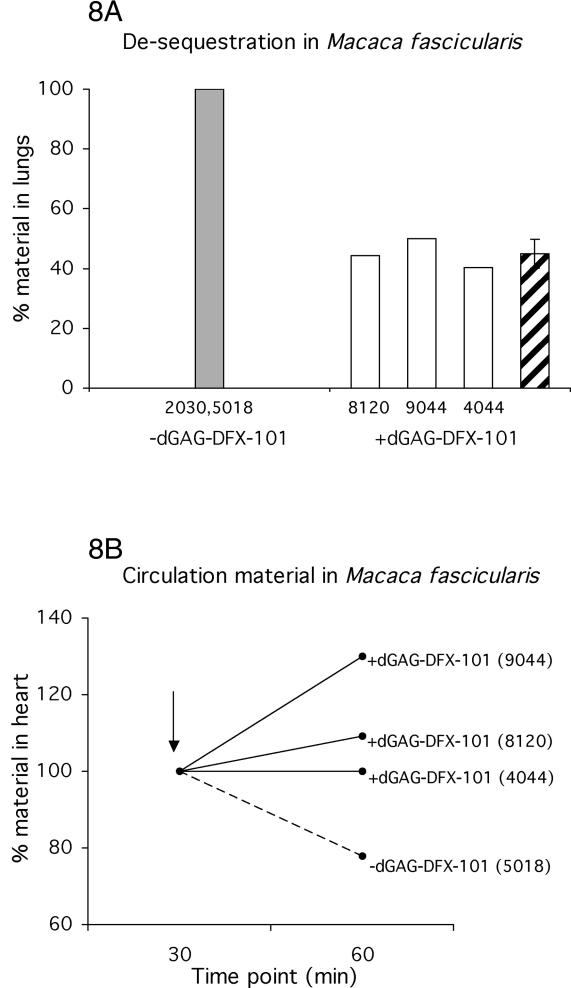
Injection of dGAG-DFX-101 Releases Sequestered P. falciparum IE into Circulation in M. fascicularis Five macaques *(M. fascicularis)* were injected with ^99m^Tc-labeled IE of the parasite FCR3S1.2 and left for 30 min. Three of the monkeys (ID 8120, 9044, and 4044) each received 500-μg dGAG-DFX-101 by intravenous injection while two monkeys (ID 2030 and 5018) were left untreated. (A) Shows the level of material in the lungs. The dGAG was injected at time point 30 min and the radioactivity in the animals were measured for 15 min 60 to 75 min after injection. The level of radioactive material in the lungs was compared with the amount of radioactive material in the whole animal. Results are presented as relative amount in lungs compared with control animal receiving no dGAG-DFX-101 (control, adjusted to 100%; grey bar). White bars show the relative amount of material in individual monkeys, and the striped bars, the mean thereof. (B) Shows the level of material in the hearts before and after the injection of the dGAG-DFX-101. The radioactive material in the hearts (i.e., in circulation) increases (9%, ID 8120; 30% ID 9044; and 0% ID 4044) after treatment with dGAG-DFX-101. In contrast, the amount of material in the heart of the monkey left untreated decreased by 22% (ID 5018). The analysis of the radioactivity in the hearts was performed by comparing amounts of material present at time point 30 min and at time point 60 min, after the dGAG-DFX-101 was injected. Time point 30 min was adjusted to 100%. The arrow shows the time point of injection of the dGAG-DFX-101.

Three macaques (ID 8120, 9044, and 4044) were injected with purified ^99m^Tc-labeled IE of FCR3S1.2, and tomography images were acquired before and after injection of the dGAG-DFX-101 at time points 30 min and 60 min. dGAG-DFX-101 (500 μg) was injected into the animal after 30 min in a total volume of 5-ml RPMI-1640 into the *Vena saphena magna*. Images at time point 30 min (immediately prior to injection of dGAG-DFX-101) revealed an average of 9.2% (9.8%, ID 8120; 9.1%, ID 9044; and 8.7 %, ID 4044) of the injected material present in the lungs, a figure similar to that of the control animal (see above, ID 5018). An average of 5.7% of the injected IE (5.7%, ID 8120; 6.0%, ID 9044; and 5.5 %, ID 4044) was left in the lungs in dGAG-DFX-101–treated animals at time point 60 min, 30 min after the injection of the dGAG-DFX-101. The corresponding figure from the lungs of the untreated animals injected with solely IE was 8.6% (see above, ID 5018 and 2030) at time point 60 min. In order to determine parasite specific sequestration of IE, the non-specific, non-PfEMP1–mediated trapping of uninfected human erythrocytes (see above, 3.4%, ID 7020) was subtracted from the amount of binding in the treated animals (5.7%, ID 8120; 6.0%, ID 9044; and 5.5 %, ID 4044) and in the untreated controls (8.7%, ID 2030; and 8.5%, ID 5018). The reduction of sequestration in the treated animals was then approximately 55% (56%, ID 8120; 50%, ID 9044; and 60%, ID 4044) when compared to untreated controls (Figure 8A). Differences in bone marrow sequestration were not sufficiently robust to be evaluated for differences between treated or untreated animals. One monkey (ID 7020) injected with ^99m^Tc-labeled IE of FCR3S1.2 was analyzed by dynamically monitoring the levels of sequestration using a two-dimensional gamma camera and X-ray before and during the treatment (unpublished data). The proportion of injected material present in the lungs over time showed a series of fluctuations directly after the injection of dGAG-DFX-101. Thereafter the lung-sequestration dropped and was stabilized at the lower level (unpublished data).

The number of ^99m^Tc-labeled IE in circulation was in all cases found to be low (<2% of injected material) as analyzed by studying the levels of ^99m^Tc-labeled IE in the heart prior to treatment (time point 30 min). However, after injection of dGAG-DFX-101 (time point 60 min), the circulating IE increased by an average of 13% (9%, ID 8120; 30%, ID 9044; and 0%, ID 4044; Figure 8B). In contrast, the amount of material present in the heart in one animal injected with IE, but left untreated, decreased by 22% (ID 5018; Figure 8B), suggesting the IE sequestration to have increased with time.

## Discussion

Here we report the generation of dGAGs for their use as adjunct treatment in patients with severe malaria. The dGAGs are derived from standard heparin; they lack anticoagulant activity, block merozoite invasion of erythrocytes, disrupt rosettes, inhibit endothelial binding in vitro, and reverse sequestration in in vivo models of severe malaria. The data suggest that the dGAGs may be useful in the treatment of severe human malaria and that the previously observed effect of heparin on cerebral malaria [22–26] is likely to depend on the antisequestration activity rather than the anticoagulant activity of heparin.

A major obstacle in the development of drugs aiming at reducing severe malaria pathology has been the lack of robust animal models for screening and evaluating drug candidates. We have therefore recently developed an in vivo model in which the sequestration (cytoadherence and rosetting) of human IE can be monitored for a short period in the rat [33]. The technique has here been further adapted for the use in macaque monkeys *(M. fascicularis)* which are intra-venously injected with ^99m^Tc-labeled human IE and then evaluated for sequestration by using a three headed gamma camera and X-ray for imaging, rendering three-dimensional information on both the distribution of the radioisotope and the anatomy of the animal (Figure 7). The technique makes it possible to avoid euthanasia while still allowing the acquisition of reliable data on the level of the sequestration of IE in vivo. The adhesion of the IE has been shown to depend on PfEMP1 in both rats and macaques by (1) vaccinating the animals with the DBL1α domain, the HS-binding fragment of PfEMP1 [33,34] (K. Moll, F. Pettersson, A. M. Vogt, C. Jonsson, N. Rasti, S. Ahuja, M. Spångberg, P. Bull, O. Mercereau-Puijalon, D. E. Arnot, M. Wahlgren, and Q. Chen, unpublished data), whereby the sequestration of the IE is inhibited, or by (2) the treatment of the animals with a PfEMP1-adhesion receptor analog [33] as also shown herein. The receptors employed by the IE of different isolates (multiadhesive, HS, CSA, CD36, or CD31 binding) have been carefully characterized in vitro [33]. The majority of the injected human erythrocytes are, however, regardless of being infected or not, absorbed by the liver, spleen, and kidneys. This reflects the incompatibility between the human erythrocytes and the animals, resulting in the presumably non-specific removal of injected material by tissue macrophages. Still, the capacity of IE to robustly adhere in the lungs of both animal species argues that these two animal models are new useful additions for the screening of antiplasmodial drugs and vaccines.

The rat model allows measurement of sequestration of human IE in the lungs of immunocompetent animals. Sequestration in the rat represents rosetting, autoagglutination, and cytoadherence [33], which is why any decrease of sequestration is likely to be a consequence of inhibition and disruption of all these adhesive events. The effect on clumping of IE with platelets is less clear. Sequestration-blocking and de-sequestration activities of the dGAGs were observed in rats both with a cloned parasite and with wild isolates, although the levels of inhibition varied somewhat. Blocking of sequestration of a cloned parasite gave a maximum effect with an intermediate quantity of dGAG-KI01, whereas higher (and lower) amounts resulted in lesser effects (Figure 5A). This trend was not as obvious while evaluating de-sequestration of the same cloned parasite in vivo (Figure 5B). The lesser response to the higher quantities of dGAGs injected might be a consequence of cross-binding between dGAG-binding molecules present on the rat endothelium, the dGAG, and the IE. Similarly, a somewhat lesser effect was also seen while inhibiting cytoadherence on rat lung sections in vitro and analyzing high concentrations of heparin and HS (Figure 3B and 3C), but this effect has not been seen with primary human endothelial cells [19].

De-sequestration by dGAG treatment was further studied in the monkeys 30 min after they had been injected with IE, when the level of sequestration was stable. Each monkey was treated with 500 μg of dGAG, resulting in a reduction of sequestration of up to 60% when comparing treated with untreated animals (Figure 8A). The effect of the dGAG on sequestration was also studied by dynamically monitoring the level of radioactive material present in the lungs before and during the treatment in one monkey (unpublished data). The results demonstrate two interesting findings: an increase in pulmonary-available parasites directly following the injection of dGAG and a later decrease in sequestration at about 10 min after the injection. The initial fluctuation may be the result of a release of previously sequestered IE from different sites in the body, resulting in a short period of elevated levels of circulating IE passing through the lungs. This interpretation is supported by the finding that the radioactive material in the heart is increased in animals injected with dGAG as compared to a reduction seen in non-treated animals (Figure 8B).

Based on the assumption that disseminated intravascular coagulation is a central mechanism in malaria pathogenesis, anticoagulant therapy with heparin was previously used with some success in the treatment of human cerebral malaria [22–26]. The use of heparin was later abandoned due to the occurrence of severe bleedings and death of some children treated [27]. However, a better knowledge of the sequestration mechanisms causing severe malaria, and recent data suggesting an important role for HS as an IE-adhesion receptor prompted us to develop novel antisequestration compounds based on heparin. The use of CSA has previously been considered for adjunct treatment of malaria [35], but parasites specific for CSA are mainly found in pregnant women and not in children or adults with cerebral malaria or respiratory distress. We therefore developed heparin analogs chemically depleted of anticoagulant activity, dGAGs, and show here that IE sequestration can be disrupted and that the de-sequestered IE are released into circulation by the injection of dGAGs. Besides the antisequestration properties, dGAGs may affect disease development by limiting the growth of parasites because dGAG inhibits merozoite invasion into fresh erythrocytes in vitro (Figure 4).

The observed effects of dGAG on sequestration probably represent not only true de-sequestration, but also blocking of initial and continued adhesion of IE that are still available in the circulation. This kind of treatment may not release 100% of the sequestered IE, although even partial de-sequestration may have an impact of the development of the disease. The sequestration of IE of some clinical P. falciparum isolates analyzed was more susceptible to the dGAGs than the sequestration of IE of other isolates. This is in concordance with previously observed differences of the dependency of HS for cytoadherence and rosetting [2,19,36]. The effects on sequestration, although sometimes relatively small, are still likely to be of fundamental importance to re-establish the circulation in the affected vasculature. The results from our in vivo experiments in rats present a therapeutic window approximately between 1–100 μg/kg, but a dose for future use in patients has to be additionally discussed and decided in view of previous human studies of heparin when used for treating severe malaria or other disease states.

The strategy of development of the dGAGs described here has yielded an effective treatment of severe malaria in two animal models. Because heparin is an endogenous molecule expressed in mast cells, immune responses are not expected to be elicited by heparin or by dGAGs, but this has to be further investigated in safety and toxicology tests. Future plans also include the development of cost-effective and stable large-scale production protocols and preclinical testing in animals and humans. If this approach is successfully translated to the clinical setting, it may offer help to patients whereby the injection of dGAGs releases already sequestered parasite-infected erythrocytes and re-establishes the micro-vascular blood flow. We suggest dGAGs to be promising candidates of adjunct therapy that may have an impact on malaria mortality. Taken together, the results presented here support the evaluation of the dGAGs as adjunct therapy in severe malaria for an impact on the mortality from this severe disease state.

## Materials and Methods

### Generation and ^3^H-labeling of dGAGs.

For the generation of heparin lacking anticoagulant activity, dGAG, the AT binding sequence within heparin was oxidized by the use of periodate, as previously described [29]. Briefly, 2-mg/ml heparin (Lövens Kemiske Fabrik, Ballerup, Denmark) was incubated in 50 mmol/l sodium citrate, 0.2 mol/l NaClO_4_ and 20 mmol/l NaIO_4_ (pH 3), at 4 °C for 2 h (KI01). The reaction was stopped by extensively dialyzing the samples against H_2_O. NaBH_4_ (20-mg/mg heparin in 1-ml H_2_O) was added to the solution and incubated for an additional 3 h at room temperature (RT) and dialyzed against H_2_O. A second dGAG batch (DFX-101) was produced in a similar manner under Good Manufacturing Practice (GMP) conditions. The preparations were freeze-dried. An aliquot of the dGAG-KI01 preparation was labeled with ^3^H by reduction with NaB^3^H, as previously described [37].

### Quantity and quality analysis of dGAGs.

Concentrations of final dGAG preparations were determined as previously described [38], and the specific activity of ^3^H-labeled KI01 was found to be 25,800 cpm/μg. The anticoagulant activity of dGAGs was tested using the APTT test Cephotest (Nycomed AB, Lidingö, Sweden). Briefly, 190 μl of human plasma mixed with different GAG preparations and 200 μl Cephotest reagent was pre-incubated for 5 min at 37 °C before adding CaCl_2_ to a final concentration of 6.7 mmol/l. Upon addition of Ca^2+^, the time was measured until clots were formed. The different dGAG preparations were compared with un-fractionated (standard) heparin, high and low AT–affinity heparin generated as described [32], and phosphate buffered saline (PBS) as negative control.

### dGAG binding to recombinant DBL1α protein and to live IE.

An in-solution binding assay of ^3^H-labeled dGAG-KI01 to DBL1α, cloned from FCR3S1.2, was carried out as described [39]. In brief, 4 μg of the recombinant fusion protein DBL1α-GST [40] or GST were incubated with different concentrations of ^3^H-labeled dGAG-KI01 in 200 μl 50 mmol/l Tris [pH 7.4], 150 mmol/l NaCl, and 0.1% BSA in duplicates for 1 h at RT. The samples were then trapped on a nitrocellulose filter (pore size 0.45 μm) and washed. Bound radioactive material was eluted with 2 mol/l NaCl and quantified by scintillation counting. The interaction between dGAG-KI01 and live IE was carried out by incubating IE (1 × 10^8^) with 0.9-μg ^3^H-labeled dGAG-KI01in RPMI for 30 min at RT with constant careful mixing. After three washes in RPMI, cells were lysed in 1 mM Tris [pH 7.4], supplemented with 0.1 mM EDTA. Samples were centrifuged, membranes were separated from supernatants, and the radioactivity was analyzed by liquid scintillation counting.

### The parasites.

The highly rosetting and autoagglutinating parasite FCR3S1.2 was obtained through cloning by micromanipulation of FCR3S1 [34]. It was cultured according to standard methods using candle jar technique [40], and the rosetting rate was kept at >75% by centrifugation on Ficoll-Paque as previously described [41]. The P. falciparum clinical isolates UAS22, UAS29, and UAS31 were obtained from children with severe malaria from Apac, Uganda (ethical permission 03/095). Informed consent was obtained from the patients and/or their guardians, 2–5 ml of venous blood was taken, and the erythrocytes were separated from the leukocytes by centrifugation on Lymphoprep (Nycomed) as described elsewhere [4] and frozen according to standard procedures [40]. For the assays, frozen blood samples were maintained and expanded in O Rh^+^ erythrocytes at 5% hematocrit with 15% AB^+^ human serum added to the malaria culture medium (MCM; RPMI-1604 supplemented with HEPES, gentamycin, and sodium bicarbonate). Gas was used to maintain a low oxygen pressure during cultivation (5% O_2_, 5% CO_2_, and 90% NO_2_; AGA, Lidingö, Sweden).

### Rosetting and cytoadherence disruption assays in vitro.

The rosetting-disruption assays with dGAGs and GAGs (heparin and HS [Lövens Kemiske Fabrik]) were performed essentially as previously described [36]. The effect of dGAG or GAGs (heparin and HS) on cytoadherence was tested using magnet-enriched IE [42], and cryosections of rat lungs as previously described [33]. IE were either added together with the dGAG to the lung sections and incubated for 30 min at 37 °C following three careful washes, or added solely to the section and allowed to adhere for 30 min at 37 °C before careful washing three times, whereafter the sections were incubated with dGAG for another 30 min at 37 °C followed by a final three washes. All washes were with RPMI-1640, and the concentrations of dGAG were as stated in the text. In both setups, samples were incubated with orbital shaking of the samples (50 rpm), and the sections were fixed in 1% glutaraldehyde (Sigma, St. Louis, Missouri, United States) in PBS at RT for 30 min before staining with Giemsa. The fixed and stained sections were analyzed in light microscopy (Nikon Optiphot, Tokyo, Japan) by counting the number of bound IE in four parallel lanes from the top to the bottom and four parallel lanes from the left to the right.

### Merozoite invasion inhibition assay in vitro.

The merozoite invasion inhibition assay with dGAGs (KI01 and DFX-101) and GAGs (heparin [Lövens Kemiske Fabrik], CSA (bovinel Fluka, Buchs, Switzerland) and HA (Streptococcus equi sp.; Fluka) was essentially performed as previously described [32]. Briefly, trophozoite-synchronized P. falciparum cultures with a parasitemia of 0.4% and a hematocrit of 2% were grown in MCM in micro-cultures (100 μl) in the presence of increasing concentrations of dGAG or GAGs at 37 °C for 24–30 h. In order to quantify the parasitemia, the samples were stained for 10 s with acridine orange and then analyzed using a FACS instrument from Becton Dickinson (Mountain View, California, United States). A minimum of 50,000 cells per sample were collected.

### In vivo studies of sequestration in rats.

Sprague Dawley rats (male, 3–6 mo old, and an approximate mean weight of 500 g; B&K, Sollentuna, Sweden) were kept in the animal facility of the Swedish Institute for Infectious Disease Control (SMI). P. falciparum–infected human erythrocytes were cultivated in vitro and enriched to a parasitemia above 70%. Prior to injection into the animals, the same number of human infected or uninfected erythrocytes were washed in RPMI and radioactively labeled with ^99m^Tc in parallel as previously described [33]. Rats were anaesthetized with Dormicum (Roche, Basel, Switzerland), Hypnorm (Janssen Pharm Centica, Beerse, Belgium), and H_2_O (1:1:2) and 0.8–2.7 × 10^7^ IE or uninfected erythrocytes, in 0.5-ml RPMI-1640, were injected intravenously into the tail vein. The treated rats were either co-injected with IE and different concentrations of the dGAG, or first injected with IE and, after 3 min, injected with different concentration of dGAG, heparin, or dextran sulfate (Leuconostoc spp.; Sigma) in 0.5-ml RPMI-1640, whereas control animals were injected with IE without dGAG, heparin, or dextran sulfate. The distribution of the labeled cells was monitored using a gamma camera for 30 min as previously described [33]. The relative amount of labeled cells sequestered in the lungs was calculated by comparing the activity of excised lungs to that of the whole animal. All the experiments were carried out with appropriate ethical permissions (N177/01, N178/01, N176/03, and N184/05).

### In vivo studies of sequestration in *M. fascicularis.*

The primate model for the study of the sequestration of P. falciparum infected human erythrocytes in macaques *(M. fascicularis)* was developed based on the rat model used herein [37]. Six female macaques (ID 5018, 2030, 8120, 9044, 4044, and 7020) were kept in the animal facility of SMI. The animals were sedated with Ketaminol Vet (Farmaceutici Gellini S.p.A, Aprilia, Italy) and during the challenge kept asleep with a combination of Ketaminol Vet and Rompun Vet (Bayer AB, Gothenburg, Sweden). Uninfected human erythrocytes or highly purified IE (>80% parasitemia) of the FCR3S1.2 clone were radioactively labeled with ^99m^Tc prior to injection into the animals as previously described [33]. Approximately 10^8^ cells were administrated by a 1-min injection of a 5-ml sample of cells diluted in RPMI-1640, into the *Vena saphena magna,* and the animals were left for 30 min to let the IE sequester. Four of the animals injected with IE (ID 8120, 9044, 4044, and 7020) were subsequently injected with 500-μg dGAG-DFX-101 per animal, diluted in 5-ml RPMI-1640, into the *Vena saphena magna* during 1 min, while two animals injected with IE (ID 5018 and 2030) and one animal injected with uninfected human erythrocytes (ID 7020) were left without additional injections. All animals, treated or untreated, were left for another 30 min. Six (ID 5018, 8120, 9044, 4044, 7020, and 2030) animals examined were analyzed by whole-body imaging in an X-ray instrument equipped with a triple-headed gamma camera allowing for three-dimensional reconstruction, both on the anatomy and the radioisotope distribution (TRIAD XLT; Trionix Research Lab, Twinsburg, Ohio, United States) of the pictures showing the anatomy of the animal as well as the number of counts in specific body volumes. The amount of injected material ending up in different organs could hence be accurately determined by careful mapping of the organs using the X-ray image and imposing this map on the image showing the radioisotope distribution. Animals analyzed with this three-dimensional reconstruction were either analyzed in the camera after 30 min and after 60 min, or only after 60 min. One animal injected with IE and treated with dGAG (ID 7020) was analyzed dynamically with a two-dimensional technique [33] for 60 min. This technique does not allow measurement of the activity in parts covered by other organs, and the absolute figures are not comparable to those acquired by tomography (three-dimensional reconstruction). After being analyzed, animals were brought back to the animal facility and kept under observation. No signs of any disorders of the animals were observed. All the experiments were carried out with appropriate ethical permissions (N308/04 and N183/05).

### Statistical analyses.

Where applicable, data are presented as mean ± standard deviation.

## Supporting Information

Figure S1 Effect of dGAG-DFX-101 on P. falciparum Rosettes and Cytoadherence of IE In Vitro (A) Aliquots of rosetting cultures (UAS22, UAS29, UAS31, and FCR3S1.2) were treated with dGAG-DFX-101 at different concentrations. The rosetting rates were counted after 30 min incubation and compared with mock-treated samples. For the cytoadherence assays (B) and (C), the IE of different P. falciparum cultures (UAS22, UAS29, UAS31, and FCR3S1.2) were allowed to attach to rat lung sections under orbital shaking (50 rpm) at 37 °C. Different concentrations of dGAG-DFX-101 were added together with IE (B) or after letting the IE adhere (C). Unbound material was removed by washes before the slides were fixed in 1 % glutaraldehyde, stained with Giemsa, and analyzed by light microscopy at a 1,000× magnification.
(34 KB PDF)

Figure S2 Effects of dGAG-DFX-101 on Reinvasion of P. falciparum Merozoites (FCR3S1.2) into Fresh Erythrocytes In Vitro Parasite culture at throphozoite stage (≈25 h of development) with a 0.4% parasitemia and a 2% hematocrit were incubated with increasing concentrations of dGAG-DFX-101 for 24–30 h at 37 °C. Levels of parasitemias were estimated using FACS counting a minimum of 50,000 cells per sample.
(27 KB PDF)

Figure S3 Injection of dGAG-DFX-101 Reduces Sequestration of P. falciparum IE in Rats (A) Rats previously administrated with ^99m^Tc-labeled IE of parasite FCR3S1.2 were injected with different concentrations of dGAG-DFX-101 for measurement of the de-sequestration effect. (B) ^99m^Tc-labeled IE of the UAS isolates (UAS22, UAS29, and UAS31) were administrated to rats simultaneously with 5 μg of the dGAG-DFX-101, whereas in (C), the de-sequestration effects of different concentrations of dGAG-DFX-101 was tested by injection of dGAG-DFX-101 3 min after the injection of IE. Rats were in all cases left in the gamma camera for 30 min after which the lungs were excised, measured for radioactivity, and compared to the radioactive material found in the whole animal. Results are presented as relative amount in lungs compared with control animal receiving no dGAG-DFX-101 (control, adjusted to 100%; grey bars). White bars show radioactivity in individual rats, and striped bars, the means thereof.
(55 KB PDF)

## Abbreviations

AT: antithrombin III
CSA: chondroitin sulfate A
DBL1α: Duffy-binding-like domain 1α
dGAG: depolymerized glycosaminoglycan
GAG: glycosaminoglycan
HA: hyaluronic acid
HS: heparan sulfate
IE: infected erythrocytes
PfEMP1: Plasmodium falciparum erythrocyte membrane protein 1
^99m^Tc: ^99m^Technetium
