# Supplementary figures and images for: Release of Sequestered Malaria Parasites upon Injection of a Glycosaminoglycan
Source: PLoS Pathog. 2006 Sep 29;2(9):e100. doi: 10.1371/journal.ppat.0020100 (PMC1579244; doi:10.1371/journal.ppat.0020100)

**Figure S1**

**A**

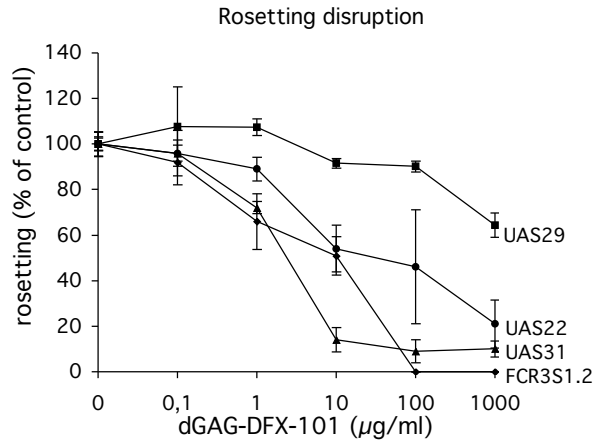

**C**

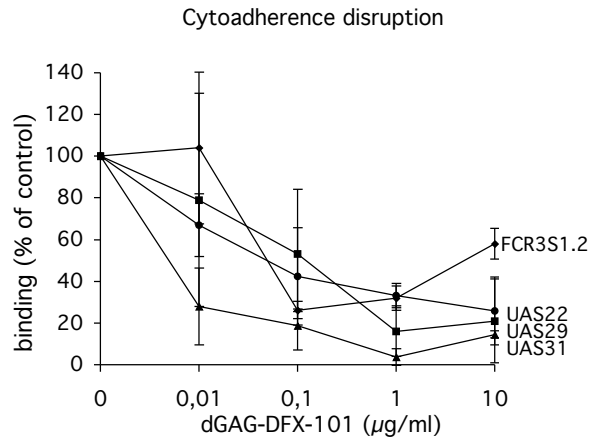

**B**

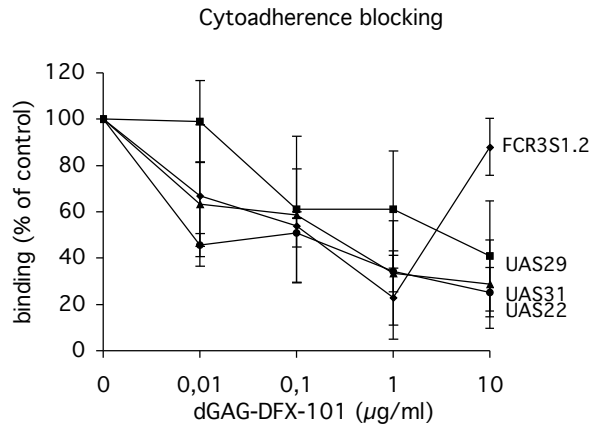

Supplement: Figure S1 — (A) Aliquots of rosetting cultures (UAS22, UAS29, UAS31, and FCR3S1.2) were treated with dGAG-DFX-101 at different concentrations. The rosetting rates were counted after 30 min incubation and compared with mock-treated samples. For the cytoadherence assays (B) and (C), the IE of different P. falciparum cultures (UAS22, UAS29, UAS31, and FCR3S1.2) were allowed to attach to rat lung sections under orbital shaking (50 rpm) at 37 °C. Different concentrations of dGAG-DFX-101 were added together with IE (B) or after letting the IE adhere (C). Unbound material was removed by washes before the slides were fixed in 1 % glutaraldehyde, stained with Giemsa, and analyzed by light microscopy at a 1,000× magnification. (34 KB PDF) [file ppat.0020100.sg001.pdf]

Figure S2

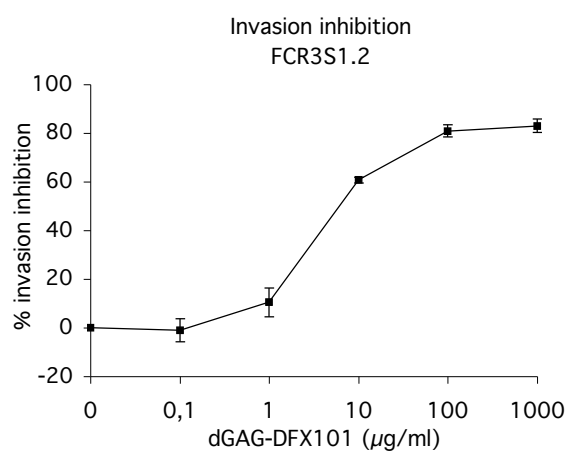

Supplement: Figure S2 — Parasite culture at throphozoite stage (≈25 h of development) with a 0.4% parasitemia and a 2% hematocrit were incubated with increasing concentrations of dGAG-DFX-101 for 24–30 h at 37 °C. Levels of parasitemias were estimated using FACS counting a minimum of 50,000 cells per sample. (27 KB PDF) [file ppat.0020100.sg002.pdf]

**Figure S3**

**A**

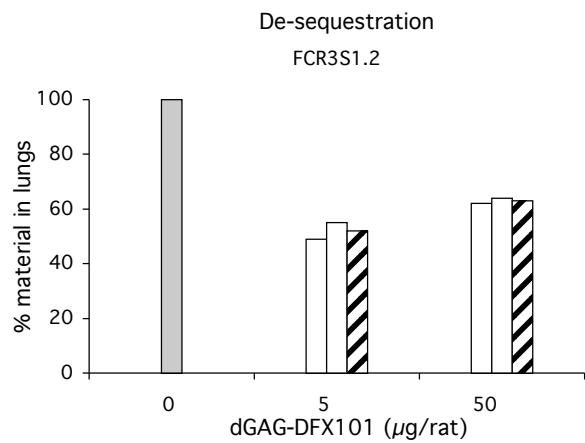

**C**

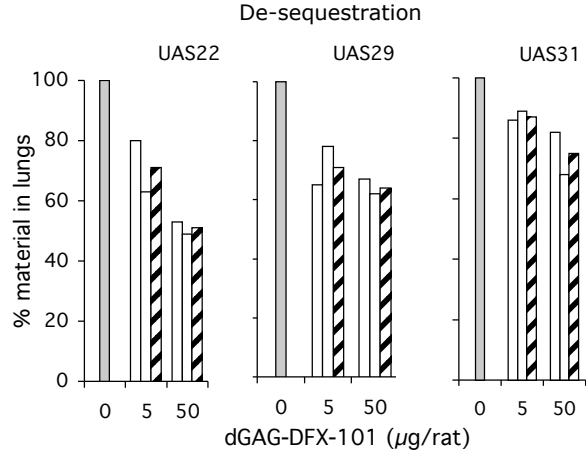

**B**

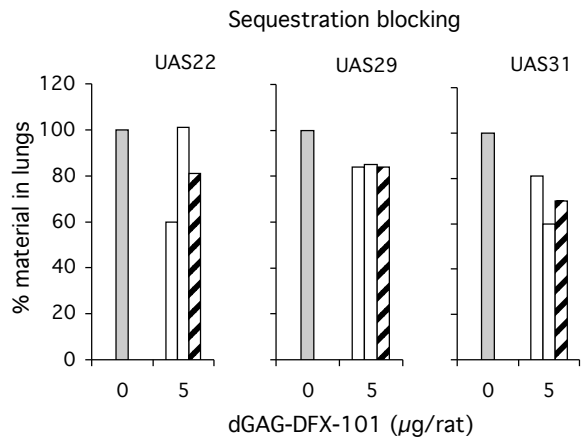

Supplement: Figure S3 — (A) Rats previously administrated with 99mTc-labeled IE of parasite FCR3S1.2 were injected with different concentrations of dGAG-DFX-101 for measurement of the de-sequestration effect. (B) 99mTc-labeled IE of the UAS isolates (UAS22, UAS29, and UAS31) were administrated to rats simultaneously with 5 μg of the dGAG-DFX-101, whereas in (C), the de-sequestration effects of different concentrations of dGAG-DFX-101 was tested by injection of dGAG-DFX-101 3 min after the injection of IE. Rats were in all cases left in the gamma camera for 30 min after which the lungs were excised, measured for radioactivity, and compared to the radioactive material found in the whole animal. Results are presented as relative amount in lungs compared with control animal receiving no dGAG-DFX-101 (control, adjusted to 100%; grey bars). White bars show radioactivity in individual rats, and striped bars, the means thereof. (55 KB PDF) [file ppat.0020100.sg003.pdf]
